# Molecular docking analysis of calcium channel blockers with ALR2 and RAGE

**DOI:** 10.6026/97320630019028

**Published:** 2023-01-31

**Authors:** Samreen Kazmi, Surekha Challa, Malini Devi Alaparthi, Indira Devi M, Swetha Sudha Nagamalla, Priya EJ

**Affiliations:** 1 Department of Biotechnology, Mahatma Gandhi University, Narketpally Mandal, Telangana, India, 508003; 2 Department of Biochemistry & Bioinformatics, GITAM University, Gandhi Nagar, Rushikonda, Visakhapatnam, Andhra Pradesh 530045; 3 Department of Genetics and Biotechnology, Osmania University, Hyderabad, India 500007

**Keywords:** Molecular docking, calcium channel, blockers, ALR2, RAGE

## Abstract

A metabolic condition called diabetes mellitus is linked to a number of substantial challenges. Advanced Glycation End Products (AGEs) and Aldose reductase (ALR2) are crucial in the slow development of several secondary complications. Selected calcium channel blockers (CCB's-1, 4-dihydropyridines) were docked against ALR2 (PDB code: 1Z3N) and RAGE (PDB code: 3CJJ) in the current study. We report that 1, 4-dihydropyridine compounds, particularly Benidipine, bind to the active sites with good efficiency. Thus, 1,4 dihydropyridine derivatives can be considered for further confirmation in drug discovery.

## Background:

Globally, people are becoming more susceptible to Diabetes Mellitus, a metabolic illness. The IDF Diabetes Atlas 10th edition 2021 estimates that there are currently 537 million individuals living with the condition, and that number is growing considerably, costing USD 966 billion [1]. Chronic diabetes causes a number of secondary complications, the majority of which are microvascular disorders [2]. These disorders are multi-factorial and are modulated by more than one pathway; inhibition one pathway activates the alternative pathways [3]. In the present study we examined few calcium channel blockers that can inhibit both ALR2 and RAGE. Aldose reductase (ALR2) of the polyol pathway plays a critical role in glucose breakdown that results in pathophysiology and vascular dysfunction. Polyol pathway leads to generation of Advanced Glycation End products (AGE's) specifically MGO (Methylglyoxal). An inflammatory response is elicited by the interaction of AGE's and Receptor for AGEs (RAGE) in vascular cells [4-5]. Various studies reported that calcium channel blockers (CCB's) in particular 1,4dihydropyridine derivatives were known to control these complications [6]. They exhibited a broad spectrum of applications in various disorders [7, 8,9,10]. Here we tested their affinity towards ALR2 and RAGE by molecular docking analysis.

## Methodology:

## Ligand preparation:

Eight CCB's with 1,4dihydropyridine group were considered to test and were downloaded from NCBI, PubChem (https://pubchemdocs.ncbi.nlm.nih.gov) . Name of the compounds along with PubChem id is given in Table 1. All the molecules were downloaded in .sdf file format and the molecules were retained in original state for further analysis. The conversion of 2D structure to 3D conformer was performed in Open Babel and the coordinates were saved in .pdb.

## Protein preparation:

The crystal structure of ALR2 (PDB code: 1Z3N) and RAGE (PDB code: 3CJJ) were downloaded from PDB data bank in .pdb format [11-12]. The protein was pre-prepared by assigning bonds, bond orders, hybridization, and by assigning charges, using Molegro Virtual Docker, CLC bio 2012, version 5.5. After pre-processing energy minimization was done and saved in .pdb format for further analysis.

## Detection of active site:

To locate the protein's active site, a thorough literature search was conducted. Additionally, using a DOG site finder based on a Gaussian filter, the volumetric and surface area characteristics of the active site were calculated [13].

## Molecular Docking:

Utilizing insilico docking with Molegro Virtual Docker (MVD), the protein-ligand interactions at the molecular level were examined. Docking analysis was performed with a grid resolution of 0.2 and a maximum of 1500 iterations [14-15]. In MVD Rerank score, a mathematical representation for ligand-protein affinity that is based on the MolDock scoring function (MolDock Score), which is derived from the Piecewise Linear Potential (PLP) scoring functions, provided the basis for the structure-based virtual screening of the compounds. Rerank score with good values for both targets was used to find the best compound with the highest affinity. Best posed compound along with interacting protein was saved in .pdb format for further analysis. Biovia Discovery studio 2021 was used to generate images at molecular level [14-15].

## Results & Discussion:

**Figure 1:**
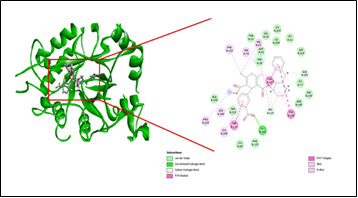
Molecular interactions of aldose reductase with benidipine

**Figure 2:**
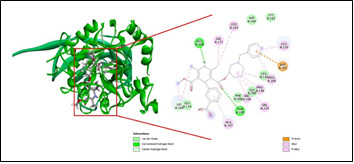
Molecular interactions of receptor for advanced glycation end products with benidipine

Drug development has emerged as the most important translational scientific technique among the research activities that contributes to the establishment of a better healthy well-being human lifestyle for use as a target therapy to treat human disorders. The discovery of better binding targets, the identification and optimization of lead compounds, preclinical trials, and phase clinical studies are all parts of drug discovery. The ultimate goal of drug development is to bring a new chemical to market that has a demonstrated therapeutic efficacy, greater binding affinity, and lower toxicity characteristics. The move from preclinical to clinical stages is a significant turning point in drug development in this setting. It consumes lot of time and money; most of the drugs failed at this stage. In order to surpass this, we made an attempt to use existing drugs to treat complications in diabetic patients by using insilico analysis. DOG site finder is used to detect active site of the proteins ALR2 and RAGE respectively. The detected cavities and their descriptors were provided in the Table 2, Table 3. All the eight compounds were tested for their affinity towards ALR2 and RAGE. The affinity of the compound against the target was established as a function of Rerank score and the data was shown in Table 4. Our results were supported by the work done by Türkescedil C *et al.* on AR2 and Matsui *et al.* on RAGE [16-17].Nivaldipine followed by Benidipine has the highest binding affinity to ALR2 of all the compounds, as can be seen from the re-rank scores with values 124.31 and 117.14 respectively. On contrary, Benidipine has highest re-rank score of 96.84 against RAGE followed by Nimodipine (re-rank score of 95.32) as shown in Table 4. To optimize Benidipine on the whole has evidenced good affinity at both the targets. Our results were supported by the work done by Matsuzaki *et al.* and Seino *et al.* [18, 19]. Figure 1 and Figure 2 depicts the ligand binding pattern of Benidipine at the active site of ALR2 and RAGE respectively along with several interactions, such as hydrogen bonding, electrostatic, hydrophobic and van der Waalinteractions, stearically that allow energetically advantageous ligand binding in the receptor.Following that, the goal was to explain why Benidipine has a superior binding profile, which may be inferred from the descriptors of receptor-ligand interactions (Table 5) that contribute energy. It is clear from the interaction energy values in the docking profile that exterior ligand interactions contribute more stability than internal ligand interactions.Comprehensively, the high binding pattern of Benidipine with ALR2 is associated with formation of hydrogen bond with Cys303, Pi-Pi stacking interaction with Tyr48, Trp111, Tyr209 and carbon-hydrogen bonds with Lys77, His110 and Gln183. It also forms van der Waals interaction with Gly18, Thr19, Lys21, Cys80, Phe115, Ser159, Asn160, Ser210, Ile260, Lys262, and Ala299. However, the high binding pattern of Benidipine with RAGE is linked to hydrogen bond formation with Glu168, Thr 195, Pi-Pi stacking interaction with Leu133, Leu159, Val194, Pro196, Ala197, Gly200, Asp201, Pro204, Val229 and van der Waals interaction with Asp160, Lys162. In addition, all selected CCB's shows good affinity towards both ALR2 and RAGE posing 1,4dihydropyridine group as an alternative pharmacophore to control these multi-factorial disorders.

## Conclusion:

We report the optimal binding of Benidipine, a 1,4dihydropyridine against both ALR2 and RAGE. This can be used as a substitute to manage diabetic secondary complications associated with hypertension.

## Figures and Tables

**Table 1:** List of calcium channel blockers selected for the present study

S. No	Name of the compound	PubChem CID
1	Amlodipine	2162
2	Barnidipine	656668
3	Isradipine	3784
4	Manidipine	4008
5	Nifedipine	4485
6	Nimodipine	4497
7	Nitrendipine	4507
8	Nivaldipine	4494

**Table 2:** Using the Active Site Prediction and Analysis Server, DoGSiteScorer, 1Z3N (Human Aldose Reductase) pockets and descriptors are provided.

Cavity number	Volume [Å³]	Surface [Å²]	Drug Score	Simple Score
P0	1059.71	106.71	0.79	0.64
P1	391.36	509.65	0.72	0.19
P2	196.29	391.6	0.38	0.02
P3	180.54	304.52	0.41	0.02
P4	146.88	215.33	0.32	0.01
P5	140.42	251.43	0.35	0

**Table 3:** Using the Active Site Prediction and Analysis Server, DoGSiteScorer, 3CJJ (Receptor for Advanced Glycation End Products) pockets and descriptors are provided.

Cavity number	Volume [Å³]	Surface [Å²]	Drug Score	Simple Score
P0	452.8	769.31	0.65	0.31
P1	340.14	583.56	0.6	0.18
P2	245.46	472.66	0.66	0.09
P3	186.15	453.42	0.33	0.04
P4	175.2	439.35	0.31	0.11
P5	159.26	381.93	0.21	0.03

**Table 4:** Molecular docking results of CCB's against ALR2 and RAGE

S. No	Name of the compound	Docking scores with ALR2 (1Z3N)		Docking scores with RAGE (3CJJ)	
		MolDock score	Rerank Score	MolDock Score	Rerank Score
1	Amlodipine	-159.06	-82.1	-123.57	-74.35
2	Benidipine	-204.53	-117.14	-156.74	-96.84
3	Isradipine	-154.82	-43.55	-133.76	-90.89
4	Manidipine	-202.77	6.36	-159.67	-94.95
5	Nifedipine	-139.72	-98.07	-112.64	-76.62
6	Nimodipine	-156.7	-62.54	-141.86	-95.32
7	Nitrendipine	-146.41	-84.62	-117.42	-76.53
8	Nivaldipine	-161.08	-124.31	-126.05	-88.33

**Table 5:** Energy overview Descriptors of Benidipine with ALR2 & RAGE

Energy Overview: Descriptors	Benidipine with ALR2 (Kcal/mol)	Benidipine with RAGE Kcal/mol
Total Energy	-206.34	-150.4
External Ligand interactions	-224.29	-140.92
Protein - Ligand interactions	-224.29	-140.92
Steric (by PLP)	-217.31	-137.8
Steric (by LJ12-6)	26.64	-23.09
Hydrogen bonds	-6.97	-3.11
Hydrogen bonds (no directionality)	-9.05	-6.19
Electrostatic (short range)	0	0
Electrostatic (long range)	0	0
Internal Ligand interactions	17.95	-10.77
Torsional strain	3.99	0
Torsional strain (sp2-sp2)	0	0
Hydrogen bonds	0	0
Steric (by PLP)	13.96	-20.26
Steric (by LJ12-6)	115.44	84.78
Electrostatic	0	0
